# Cloning and over expression of non-coding RNA rprA in E.coli and its resistance to Kanamycin without osmotic shock

**DOI:** 10.6026/97320630013021

**Published:** 2017-01-31

**Authors:** Azita Sahni, Mohammadreza Hajjari, Jamshid Raheb, Ali Mohammad Foroughmand, Morteza Asgari

**Affiliations:** 1 Nour Danesh Institute of Higher Education, Department of Biology, Isfahan, Iran;; 2 Department of Genetics, Faculty of Science, Shahid Chamran University of Ahvaz, Ahvaz, Iran;; 3 National Institute of Genetic Engineering and Biotechnology, Tehran, Iran;

**Keywords:** Escherichia coli, non-coding RNA, rprA, Kanamycin

## Abstract

Recent reports have indicated that small RNAs have key roles in the response of the E.coli to stress and also in the regulating of virulence factors. It seems that some small non-coding RNAs are involved in multidrug resistance. Previous studies have indicated that rprA can increase the tolerance to Kanamycin in RcsB-deficient Escherichia coli K-12 following osmotic shock. The current study aims to clone and over-express the non-coding RNA rprA in E.coli and investigate its effect on the bacterial resistance to Kanamycin without any osmotic shock. For this purpose, rprA gene was amplified by the PCR and then cloned into the PET-28a (+) vector. The recombinant plasmid was transformed into wild type E.coli BL21 (DE3). The over expression was induced by IPTG and confirmed by qRT-PCR. The resistance to the kanamycin was then measured in different times by spectrophotometry. The statistical analysis showed that the rprA can increase the resistance to Kanamycin in Ecoli K12. The interaction between rprA and rpoS was reviewed and analyzed by in silico methods. The results showed that the bacteria with over-expressed rprA were more resistant to Kanamycin. The present study is an important step to prove the role of non-coding RNA rprA in bacterial resistance. The data can be the basis for future works and can also help to develop and deliver next-generation antibiotics.

## Background

Bacteria must constantly adapt to the external and environmental conditions by adjusting their physiology and behavior. It appears that the phenotype of bacterial resistance to antibiotics is frequently regulated through some biochemical pathways. The prevalence of antibiotic resistance in infections caused by E.coli is high [1,2,3]. “Small non-coding RNAs” (sRNAs) are molecules, which have gained much attention in recent studies due to their potential roles in antibiotic resistance [4]. The reports have indicated that sRNAs have key roles in the response to different stresses and regulation of bacterial virulence factors [5]. The first Small non-coding RNA regulator in E.coli was named MicF RNA, which has 174 nucleotides. This small non-coding RNA inhibits the translation of ompF mRNA encoding the major outer membrane purine [6]. This discovery persuaded other researchers to identify small non-coding RNAs and find their characteristics in a variety of bacteria with different methods [6]. In bacteria, non-coding RNAs (ncRNAs) are regulated by different ways. The RNAs can affect the function of the protein, transcription initiation, stability, and initiation/elongation of mRNA translation. NcRNA transcripts are categorized into two groups including anti sense RNAs (asRNAs) and trans encoded RNAs. The asRNAs are fully complementary for mRNA targets, while trans-encoded sRNAs are much shorter than their complementary regions [7].

RprA is a small non-coding RNA with 106 nucleotide length. It seems that rprA forms three stem-loop structures and is highly conserved. It is also suggested that rprA contributes in the positive regulation of RpoS translation. RprA promoter is positively regulated by RcsB [8], which is a capsule synthesis response regulator. Under stressful stimulus signal, RcsB is activated and so increases the rprA expression. Then, RprA expression seems to increase the translation of RpoS [9]. Amirault et al. reported the importance of this gene in Kanamycin resistance in E.coli after osmotic shock [10]. Since it is important to analysis the effect of rprA in different conditions, the current study was designed to clone and over express this gene in E.coli. In order to find the role of rprA in different conditions, we tried to analyze the overexpression of rprA in the wild type Bl21 E.coli in different times. In the current study, we analyzed this effect by pET expression system and without any osmotic shock. Also, we tried to analysis and review the interaction of rpoS-rprA RNAs through in silico methods. The results can provide a context to prove the role of the rprA in antibiotic resistance, which can be a basis for future works. It can also help to develop and deliver next-generation antibiotics.

## Methodology

### Gene Amplification

After Bacterial DNA extraction by boiling method, the rprA gene was amplified using polymerase chain reaction (PCR) through specific primers (Table 1). PCR process was performed under the following conditions: The initial denaturation step at 94 °C for 3 minutes, then 35 consecutive cycles of denaturation steps at 94 °C for 30 seconds, annealing at 58 °C for 30 seconds, extension at 72 °C for 30 seconds, final extension for 10 min at 72 °C. Finally, the PCR product was loaded on 1% agarose.

### Cloning of rprA and over-expression in E.coli

The PCR product and pET 28a plasmid were digested by Xba1 and HindIII enzymes (Fermentas Company). The products were loaded on the gel and purified by gel extraction kit (GENALL Company). RprA gene was cloned into the pET28a plasmid using T4 ligase enzyme (Sinaclone) and the resulting recombinant plasmid was called pET28a/rprA. Recombinant plasmid was sequenced to verify the gene insertion. Recombinant plasmid was transformed into the competent E.coli BL21 (DE3) by chemical methods using heat shock [11]. The transformed bacteria were then cultured in Luria-Bertani medium (LB) containing kanamycin (30μg/ml) in shaker incubator at 37 ºC with 200 rpm for 2 hrs. When the OD reached to 0.4-0.6, the induction was done by IPTG at a final concentration of 0.5 mM. The culture was again continued with previous condition, and sedimentation was performed 6 hours after the induction with centrifugation at rpm4000 for 4 minutes.

### RNA extraction, cDNA synthesis, and RT-PCR

The expression analysis was done by Reverse Transcriptase PCR method. The RNA was extracted using Sina pure kit and the cDNA was synthesized from colonies by Omni script reverse transcription kit. DNAase was used in order to remove any genomic DNA. Semi quantitative RT-PCR reaction was finally performed using the cDNA as template. 16srRNA was also used as endogenous control in RT-PCR (Primers are in Table 2).

### Study of bacterial resistance to Kanamycin

To check the bacterial resistance to kanamycin, the new Luria- Bertani (LB) medium was made and divided into the equal amounts. Kanamycin and overnight-cultured recombinant bacteria were equally added into each falcon tube containing the medium and placed into the shaker incubator at 200 rpm at 37 ºC for 2 hrs. When the OD reached to about 0.5, the IPTG with the final concentration of 0.5 mM was added into the falcon tubes assigned as “over-expressed bacteria”, whereas the remaining tubes were not treated with IPTG (assigned as controls). Cultures were then incubated and culture turbidity was read after 2, 4, 6, and 8 hours [10,12].

### Statistical analysis

The data for growth of bacteria were analyzed by spectrophotometry. The difference between growth of over expressed bacteria and control bacteria was analyzed by two-way analysis of variance (ANOVA) method through Graphpad Prism5 software.

### In silico prediction of RNA-RNA interaction and folding energy

The EcoCyc database (ecocyc.org) was used for describing the structure of rpoS operon and interactions. Then, the sequence of rpoS (from the transcription start site 4) was retrieved from the Genebank. The interaction between rpoS AND rprA RNAs was analyzed by IntraRNA program (rna.informatik.unifreiburg. de/). The hybridization energy was calculated through this software.

## Results

**Figure 1:**
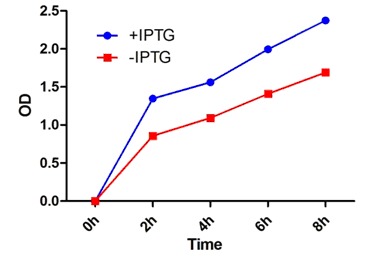
Comparison of the growth rate between overexpressed bacteria and negative control bacteria with LB and Kanamycin culture. The lines show the OD of two groups of bacteria (+IPTG : rprA overexpressed; -IPTG: Non over expressed bacteria) in different times. Two Way ANOVA shows the significant difference between growth rate of two groups.

**Figure 2:**
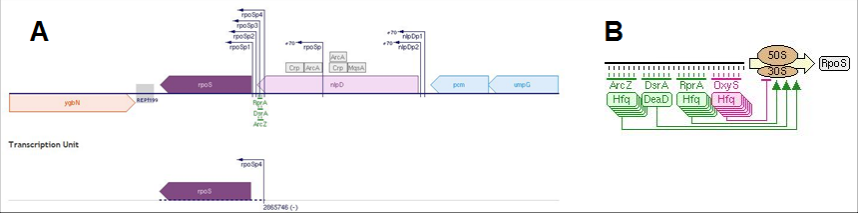
(a) Schematic representation of rpoS operon in E.coli, (b) Schematic representation of the effect of rprA on the translation of rpoS. The images are achieved from EcoCyc database.

**Figure 3:**
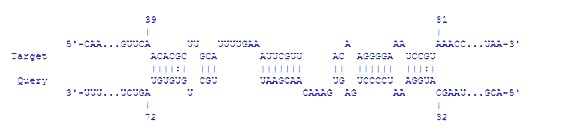
The interaction between rpoS (Target) and rprA (Query) RNAs. The data are drawn from IntraRNA software.

After the cloning, the accuracy of pET28a/rprA recombinant plasmid was examined. The screening of the colonies was done by direct PCR and sequencing. After the IPTG using RT-PCR with 16srRNA as the endogenous control performed induction the expression analysis at the RNA level. The analysis showed that the overexpressed bacteria have the higher amount of rprA level compared to the control bacteria. The spectrophotometry and statistical analysis showed that the growth rate of overexpressed bacteria is increased compared to control bacteria (Pvalue< 0.001) in different times after the IPTG treatment (Figure 1). The results show that the overexpression of rprA can increase the resistance to Kanamycin in E.coli. Furthermore, we also checked the interaction of rprA and rpoS through EcoCyc. We got the operon structure and the mechanism of action of RNA-RNA interactions (Figure 2 a, b ). The sequence of the rpoS RNA transcribed from the transcription start site4 was retrieved from GeneBank. Then, the RNA-RNA interaction between rprA and rpoS was analyzed and reviewed by IntraRNA software (Figure 3). The analysis showed that the energy is about -16.63340 Kcal/mol. The details of the interaction are depicted in Figure 3.

## Discussion

E. coli usually grows slowly in response to changing environmental conditions due to the limitations of food sources needed to protect and survive under stress conditions. To survive in such difficult conditions, E.coli expresses multiple genes involved in both transient and long-term emergency responses. Many of these genes depend on stress / stationary phase sigma factors, namely Rpos, for their transcription [12]. RpoS translation increases quickly following the stress stimuli. In this response, Hfq, and sRNAs such as DsrA and rprA are involved in the activation of rpoS translation. These two sRNAs can activate the RpoS translation by pairing with rpoS mRNA with the help of Hfq [13]. So, rprA is one of the small non-coding RNAs, whose expression increases the RpoS translation. These interactions are reviewed in the Figures 2 and 3. In general, when RprA is produced in high levels, it can activate σS (RpoS) synthesis by stabilizing the rpoS mRNA [14,15]. Amirault et al. recently showed that rprA expression, a regulator for RpoS translation, increases resistance to kanamycin in Escherichia coli K-12 following osmotic shock. They showed that rprA expression from inducible arabinose promoter can increase minimum inhibitory concentration (MIC) in ΔrcsB cells [10]. Although the statistical analysis was not performed on their data, they concluded that it can be some rprA-independent factor missing from the ΔrcsB strain which increased the tolerance of wild type cells to Kanamycin. In this study, by statistical analyses, we also showed that the resistance to Kanamycin could be increased without any osmotic shock in Ecoli K12 with inducible beta galactosidase promoter and without any osmotic shock.

Some conditions such as osmotic shock can induce the general stress response genes through the central regulator RcsB. Previous studies have indicated that the increased susceptibility to kanamycin in rcsB deficient strain was because of the inability to produce extracellular capsule. Amirault et al. investigated the role of rprA in restoring kanamycin resistance of E. coli after osmotic shock. They used the rcsB knockout strain transformed with a plasmid containing rprA under an arabinose-inducible promoter. ΔrcsB mutants are unable to form a capsule; this result may have been due to an antibiotic-resistance effect conferred by capsule presence [16,17]. In this study, we aimed to confirm the effect of rprA without any external stress such as osmotic shock. This can support the independency of the function of rprA in kanamycin tolerance. We studied this effect through pET-28 vector system, which has the kanamycin resistance gene itself. The statistical analysis confirm the results of Amirault and colleagues. These results can be the fundamental knowledge, which can be used to develop and deliver the next generation of antibiotics.

## Conflicts of interest

None

## Figures and Tables

**Table 1:** The primers used for amplification of rprA gene from E.coli for cloning into PET28 vector.

Primer	Fragment Length	Enzyme cutting sit	Primer Sequence
rprA-F	106	Xba1	CTAGACTCTAGAACGGTTATAAATCAAC
rprA-R	106	HinDIII	GTTGACAAGCTTAAAAAAAAGCCCATCG

**Table 2:** The primers used for expression analysis of rprA by RT-PCR.

Primer	Fragment Length	Primer Sequence
RT-rprA- F	78	5’ATGGAAATCCCCTGAGTG3’
RT-rprA-R		5’AAAAGCCCATCGTGGGAG3’
16SRNA-F	230	5’AGTACTTTCAGCGGGGAGGA3’
16SRNA-R		5’CGAGACTCAAGCTTGCCAG3’
